# The impact of the single exit price policy on a basket of generic medicines in South Africa, using a time series analysis from 1999 to 2014

**DOI:** 10.1371/journal.pone.0219690

**Published:** 2019-07-31

**Authors:** Rajatheran Moodley, Fatima Suleman

**Affiliations:** 1 Discipline of Pharmaceutical Sciences, School of Health Sciences, University of KwaZulu-Natal, Durban, South Africa; 2 Faculty of Sciences, Utrecht University, Utrecht, The Netherlands; University of the Western Cape, SOUTH AFRICA

## Abstract

**Background:**

Regulating pharmaceutical markets have become a key strategy by most governments in ensuring the availability and accessibility of quality medicines to its citizens. The South African government, when faced with high medicine prices, implemented the Single Exit Price (SEP) in 2004. This study assessed the impact of the of the Single Exit Price (SEP) regulation introduced in South Africa in 2004 on a basket of generic.

**Method:**

Private sector price data of a basket of medicines (December 1999 to December 2014) was obtained from various price files (Pharmacy Software Vendors and Community Pharmacy). The price of the medicine was expressed in a single unit dose. The medicines investigated used the WHO/HAI methodology. The Interrupted Time-Series (ITS) model was used to estimate the change in slope and level of medicines investigated (50 originator and its available generics) before and after the policy change.

**Results:**

Majority of the medicines analysed reflect a substantial decrease in medicine prices immediately after implementation of the pricing regulations as reflected in both the change in level and the change in slope using the interrupted time series analysis.

**Discussion:**

This study indicates that the SEP regulation had an impact on medicine pricing in South Africa in both the short (immediately on the introduction) and long term (over the study period). Most medicines investigated showed a smaller yearly increase in price compared to before regulations.

**Conclusion:**

This study provides evidence of the impact of medicine pricing intervention from a middle–income country, and useful lessons can be drawn by other developing countries looking at introducing medicine price controls.

## Introduction

In South Africa, as in many parts of the world affordability is a barrier to gaining access to quality pharmaceutical therapies.[1] Regulating pharmaceutical markets is one method used by policy makers to achieve savings.[2] Carone, et al. indicated that promoting the use of generic medicines was one cost-effective attempt at price control and containment.[2] Other methods include creating a transparent pricing system for medicines (a key strategic imperative of the South African National Department of Health (NDoH)), regulating reimbursement for dispensers, controlling wholesale and intermediaries margins, and fixing and publishing the manufacturer price of medicines. More complex methods include health-technology assessments to ensure cost-effectiveness of new pharmaceuticals, and rational use of medicines to control public budgets.[3]

With the introduction of democracy in 1994 the new South African government was faced with high medicine prices in the private sector, which included a 29.9% (1994) spend of all claims reimbursed on medicines.[4] In 1996 the Government introduced the National Drug Policy outlining among other policies, the intention to establish a pricing committee to regulate medicine prices, create transparency in the pricing structure from manufacturer, wholesaler, distributor and providers of service, as well as to ensure a non-discriminatory pricing system.[5] The Medicines and Related Substance Control Amendment Act 90 of 1997, implemented on 2 May 2003, banned the offer of discounts and rebates to patients and healthcare providers (bonusing section 18G) and establishing a pricing committee (section 22G). [6] The pricing committee made recommendations to the Minister of Health to implement the Single Exit Price (SEP) in 2004, effectively moving the private sector from a free market to a regulated environment.[7] The components of the single exit price include the ex-manufacturer price combined with the logistics fee (as determined by the manufacturer) and Value Added Tax (VAT). [8] The SEP for each medicine in the market in 2004 was a mandatory declaration of the weighted average of all 2003 sales after taking into account all discounts and off-invoice rebates. [9] Further, the 1997 amendments to the Medicines and Related Substances Act in terms of section 18A, prevented pharmaceutical manufacturers from offering discounts and or rebates. [7]

The SEP is the only price available in the private sector across the country before the addition of the regulated dispensing fee to the end user or patient. There is an annual regulated adjustment and the regulation applies to all registered medicines and Schedule substances as per the Medicines Act except those classified in the Schedule zero category which has been specifically exempted by the Minister from the pricing regulations. The SEP regulation excludes the Government or public sector where a tender process applies.

Sound pharmaceutical policies contribute to a country’s socio-economic development and the country needs economic growth for healthcare systems to perform well. [10] This further requires strategic long-term planning, effective regulations to ensure minimizing inefficiencies and unnecessary mark-ups in the supply chain and best possible pricing models to ensure access. Policy makers are aware that savings can be achieved with generic medicines, without compromising quality, if pharmaceutical markets are properly regulated.[2]

It is suggested that countries need to examine their regulatory framework and look at trends that may limit the potential for savings by inadvertently encouraging higher priced generics.[11] Seeley and Kanavos stated that the number of generic entrants is not a predictor of lower generic prices, but the market does need a significant number of entrants to impact the competition.[11] Countries like Canada, also cap the price at which generics enter a market as a policy option. Canadian provinces followed the Alberta model for pricing of generic drugs, a model suggested by academics Cambourieu, et al. [12]; Hollis,[13]; Hollis and Grootendost,[14]. In April 2014, Alberta introduced their generic policy where new generic entries start at 70% of the brand if there is only one generic entrant, and then subsequent generic medicines that entered the market were priced at 50%, 25% and 18% respectively of the originator. [14] The first generic entrant keeps the advantage for one year after which the 50% price applies. The savings through agreements with manufacturers using this model is expected to be $3.8 billion over three years to all payers.

Hassali, et al. recommended that the main policy to promote generic medicines needs to be supported by complementary policies both to facilitate its implementation and also to overcome the barriers that hinder its effectiveness. [15] The policies promoting generic substitution is seen as means to contain pharmaceutical expenditures and are often at the forefront of yielding significant cost saving. [2] Very little is known about using pricing policies as a means to contain generic medicine prices. South Africa has done so, yet no published information exists regarding the impact of pricing policies that were implemented post democracy.

### Aim of the study

The aim of the study was to examine the impact of the regulatory change, the SEP, on a basket of generic medicines from 1999–2014. The study went further to compare the difference in impact to the basket of the originator and generic medicines.

## Methodology

The interrupted time series (ITS) was used in this longitudinal pharmaceutical policy evaluation method in studying the impact of legislative changes on pricing of generic medicines in the South African private market. A quantitative analytical approach was used in this study. [16] Longitudinal trends were compared before and after the introduction of policy changes. The research tracked annual price changes on a basket of products five years before regulatory changes (1999–2003) and then measured annual price changes over the next ten years (2004–2014), following the intervention, namely, the Single Exit Price (SEP) of medicines.

The segmented linear regression was used for the interrupted time series analysis (ITSA). This divides the time series into pre- and post- intervention segments. As the regulations were introduced in 2004 (a year after the Act) and became immediately implementable, 2004 was chosen as the intervention between segments. The linear regression model has two parameters, the change in level and the change in slope. The difference between the two segments was quantified by testing the change in these two parameters. It must be noted that the pricing intervention was scheduled to come into effect one year after promulgation of the Act, hence the use of 2004 as the intervention point with data being collected on the 31 December of each year.[17]

Commonly used data source for time series is cost data obtained from pharmacy dispensing files, claims data, and other routinely collected data. SEP prices of medicines listed were obtained from the computer vendors responsible in maintaining price files for pharmacy and verified through the pharmacy dispensing systems spanning the period 1999 to 2014. The Government medicine price database[18], was created after the introduction of the SEP, and only exists post the intervention and therefore could not be utilized. It was also important to utilize a single complete data source to ensure accuracy of results.

Pricing data for the medicines being studied could not be obtained before 1999 in the country and was identified as a limiting factor. Stata (13 MSI) (StataCorp LP, College Station, TX, USA), a statistical package was used to analyse the data, generate the necessary variables, compute the statistical analysis and produce the necessary graphs. [19] To ensure unbiased estimation, stationarity and autocorrelation were taken into account as observations over time are correlated. Autocorrelation and stationarity were therefore tested and corrected for, if present, using autoregressive moving average (ARIMA) models. The following formula was used to calculate the limits used to define outliers in the data set for each of the three categories:

Upperlimit:Q3+(IQRx1.5)

Lowerlimit:Q1–(IQRx1.5)

Anything outside of the calculated limits was identified as an outlier and excluded from the data set. Once the outliers were excluded, descriptive statistics were performed on the three data sets including calculations of the mean, standard deviation, and inter-quartile range (IQR). The descriptive statistics are presented in boxplots.

### Selection of the basket of products

A basket of fifty (50) originator medicines were chosen implementing the WHO/HAI methodology. [20] The maximum number of generic molecules were chosen with a history of pricing from 2004 resulting in at least three to four generics per originator medicine with a total of 136 generic medicines being examined.

The Global Core of fourteen items (14) originator and forty six (46) generics allows for international comparison, a Regional Core of fifteen (15) originator and forty two (42) generics items allows for regional differences in medicine usage whilst still enabling comparison across countries and the twenty-one (21) originator and forty eight (48) generic medicines from a supplementary list selected for their local importance [20] completed the basket. The May 2016 update on the WHO/HAI [21] methodology removed the Regional Core and replaced it with thirty-six (36) medicines. This study used the original method as the investigation period covered 1999–2014. Further, since the regulations affected mainly the private sector in South Africa, an assessment of the top 50 medicines dispensed by volume in the private sector (IMS Health) in 2014 was taken into consideration. This data was sourced from IMS Health and used in the supplementary list. Consideration was also given to the list used in the 2004 study by Xiphu and Mpanza [22] for further comparison. Once the 50 medicines were selected, the originator and generic product was listed together with the strength, form, pack-size and National Pharmaceutical Product Index (NAPPI). The NAPPI code is a unique coding system used in South Africa. This allowed ease of reference when pricing was compared from different data files. Any price change listed on the data file in December of each year was captured.

## Results

The results of the interrupted time-series analysis (ITSA) for three groups of medicines listed as Global Core, Regional Core and Supplementary are presented in Tables 1, 2 and 3 respectively. The total number of molecules included in the three baskets were 186. Of this, 65 molecules had insufficient data either being withdrawn before the end of the study or introduced later in the study period.

**Table 1:** Global core interrupted time-series analysis for originator and generic molecules using pricing data from 1999 to 2014 with 2004 as the interruption in the series (P < 0,05).

INN	Trade names (Originator in bold)	Trend	(P value)	Change in level	(P value)	Change in slope	(P value)	Constant	(P value)	Int 1	*% Change in level 2004*
**Salbutamol 2mg/5mls Syr**	**1, Ventolin**	0,018	**(0,000)**	**-0,065**	**(0,000)**	**-0,014**	**(0,000)**	0,19	**(0,000)**	0,28	-23,47
(53)*	51, Asthavent	-0,001	0,677	-0,004	0,358	**0,005**	**0,002**	0,08	**(0,000)**	0,08	-4,64
	52, Venteze	0,007	**(0,000)**	**-0,031**	**(0,000)**	**-0,004**	**0,005**	0,07	**(0,000)**	0,11	-28,97
**Glibenclamide 5mg tab**	**2, Daonil**	0,228	**(0,000)**	**-0,771**	**0,001**	-0,047	0,382	2,45	**(0,000)**	3,59	-21,51
	54, Glycomin	0,116	0,011	**-0,764**	**(0,000)**	**-0,171**	**0,001**	0,86	**(0,000)**	1,44	-53,24
	55, Bio-Glibenclamide	-0,001	0,939	**-0,192**	**0,001**	0,007	0,557	0,33	**(0,000)**	0,32	-59,26
	56, Sandoz-Glibenclamide	0,150	**(0,000)**	**-1,436**	**(0,000)**	**-0,141**	**(0,000)**	0,82	**(0,000)**	1,57	-91,52
**Atenolol 50mg caps**	**3, Tenormin**	0,427	**(0,000)**	**-1,242**	**(0,000)**	**-0,209**	**0,007**	2,56	**(0,000)**	4,70	-26,45
** **	57, Hexa Bloka	0,053	**(0,000)**	**-0,910**	**(0,000)**	**-0,025**	**0,01**	1,07	**(0,000)**	1,34	-68,01
** **	58, Sandoz-Atenolol	0,013	0,442	**-0,849**	**(0,000)**	0,008	0,644	1,14	**(0,000)**	1,20	-70,75
** **	59, Ten Bloka_2	0,042	0,005	**-0,681**	**(0,000)**	0,001	0,912	1,13	**(0,000)**	1,34	-50,90
**Captopril 25mg tabs**	**4, Capoten**	0,044	0,014	-0,117	0,071	0,016	0,365	2,09	**(0,000)**	2,31	-5,07
** **	60, Zapto	0,006	0,308	**-0,628**	**(0,000)**	0,005	0,394	0,84	**(0,000)**	0,87	-72,52
** **	61, Capto-Hexal	-0,164	**(0,000)**	**-0,309**	**0,002**	**0,183**	**(0,000)**	1,41	**(0,000)**	0,59	-52,02
** **	62, Mylan-Captopril	-0,052	**(0,000)**	**-0,612**	**(0,000)**	**0,061**	**(0,000)**	1,00	**(0,000)**	0,74	-82,37
**Simvastatin 20mg tabs** (63,64,65)*	**5, Zocor**	-0,997	0,001	-1,078	0,225	**0,832**	**0,004**	9,56	**(0,000)**	4,58	-23,54
**Amitriptyline 25mg tabs**	**6, Tryptanol**	0,176	**(0,000)**	**-0,397**	**(0,000)**	**-0,169**	**(0,000)**	1,70	**(0,000)**	2,58	-15,42
(68)*	66, Trepiline	0,115	**(0,000)**	**-0,715**	**(0,000)**	**-0,094**	**(0,000)**	0,66	**(0,000)**	1,23	-57,94
** **	67, Sandoz Amitripyline	0,081	**(0,000)**	**-0,696**	**(0,000)**	**-0,072**	**(0,000)**	0,71	**(0,000)**	1,11	-62,59
**Ciprofloxacin 500mg tabs**	**7, Ciprobay**	-1,028	0,002	**-5,113**	**(0,000)**	**1,560**	**(0,000)**	18,21	**(0,000)**	13,07	-39,12
(69,71)*	70, Cifloc	-5,526	**(0,000)**	***1*,*672***	**(0,000)**	**5,603**	**(0,000)**	16,24	**(0,000)**	***-0*,*34***	***-499*,*10***
	72, Cifran	-5,122	**(0,000)**	***2*,*556***	**0,003**	**4,927**	**(0,000)**	15,60	**(0,000)**	***0*,*24***	***1078*,*48***
**Co-Trimoxazole 8+40mg/ml syr**	**8, Bactrim**	0,364	**(0,000)**	**-2,267**	**(0,000)**	**-0,247**	**(0,000)**	2,46	**(0,000)**	4,28	-52,94
(75)*	73, Purbac	0,051	**(0,000)**	**-0,490**	**(0,000)**	**-0,040**	**(0,000)**	0,36	**(0,000)**	0,61	-80,20
	74, Cozole	0,051	**(0,000)**	**-0,490**	**(0,000)**	**-0,040**	**(0,000)**	0,36	**(0,000)**	0,61	-80,20
	76, Adco-Co-Trimoxazole	0,057	**(0,000)**	**-0,426**	**(0,000)**	**-0,053**	**(0,000)**	0,32	**(0,000)**	0,61	-70,30
**Amoxicillin 500mg caps**	**9, Amoxil**	0,334	**(0,000)**	-0,127	0,429	**-0,274**	**0,001**	3,52	**(0,000)**	5,19	-2,45
	77, Moxymax	0,062	**(0,000)**	**-1,331**	**(0,000)**	**-0,051**	**0,001**	1,36	**(0,000)**	1,67	-79,56
	78, Betmox	0,011	0,5	**-1,277**	**(0,000)**	0,007	0,666	1,58	**(0,000)**	1,64	-77,91
	79, Zoxil	0,017	0,827	**-0,894**	**0,008**	-0,022	0,781	1,73	**(0,000)**	1,81	-49,31
**Ceftriaxone 1g/vial inj**	10, Rocephin	4,302	0,081	**-82,503**	**(0,000)**	-3,371	0,237	121,81	**(0,000)**	143,32	-57,57
(80,81,82)*	83, Aspen Ceftriaxone	1,444	0,456	**-82,325**	**(0,000)**	-1,974	0,328	117,15	**(0,000)**	121,48	-67,77
**Diazepam 5mg**	11, Valium	0,318	**(0,000)**	**-0,772**	**(0,000)**	-0,213	**(0,000)**	1,00	**(0,000)**	2,59	-29,83
(86)	84, Pax	0,003	0,376	**-0,063**	**(0,000)**	0,003	0,457	0,13	**(0,000)**	0,15	-42,86
	85, Betapam	0,003	0,064	**-0,057**	**(0,000)**	0,001	0,685	0,09	**(0,000)**	0,11	-54,29
**Diclofenac 50mg tabs**	12, Voltaren	0,063	0,013	**-0,209**	**0,025**	0,021	0,373	1,16	**(0,000)**	1,48	-14,17
(88)*	87, Diclohexal	0,071	**(0,000)**	**-1,002**	**(0,000)**	-0,053	**(0,000)**	1,05	**(0,000)**	1,41	-71,32
	89, Panamor	0,043	**(0,000)**	**-1,119**	**(0,000)**	-0,032	**(0,000)**	1,16	**(0,000)**	1,38	-81,15
**Paracetamol 25mg/ml syr**	13, Panado	0,001	0,702	**-0,030**	**0,017**	0,014	**(0,000)**	0,18	**(0,000)**	0,18	-16,57
(91)*	90, Napamol	0,006	0,272	**-0,092**	**(0,000)**	-0,004	0,471	0,09	**(0,000)**	0,12	-74,80
	92, Painamol	0,001	0,777	**-0,018**	**0,275**	0,006	0,273	0,07	**(0,000)**	0,07	-25,35
	93, Calpol	0,012	0,15	**-0,037**	**0,22**	0,002	0,782	0,14	**(0,000)**	0,20	-18,50
Omeprazole 20mg tabs(94,95,96)*	14, Losec	-0,610	0,036	**1,183**	**0,245**	1,298	**(0,000)**	11,58	**(0,000)**	8,53	13,87

Notes

Int 1(Estimate in 2004) = Cons + (Trend X Years)

Statistically Significant (P<0,05) in BOLD

Global Core List with Data (Originals: 14, Generics: 29) = 43

Change in level = 36 (83,72%)

Change in slope = 28 (65,12%)

Global Core List with no data * = 17

Global Core List Total = 60

**Table 2:** Regional core interrupted time-series analysis for originator and generic molecules using pricing data from 1999 to 2014 with 2004 as the interruption in the series. Statistically significant values (P < 0,05).

INN	Trade names (Originator in bold)	Trend	(P value)	Change in level	(P value)	Change in slope	(P value	Constant	(P value)	Int 1	*% Change in level 2004*
**Albendazole 200mg tabs**	**15, Zentel**	0,571	0,002	**-2,812**	**(0,000)**	**0,74**	**0,001**	12,272	**(0,000)**	15,127	-18,59
(98)*	97, Bendex	0,379	0,09	**-2,703**	**0,001**	0,139	0,527	9,638	**(0,000)**	11,154	-24,23
**Amlodipine 5mg tabs** (99,100,101)*	**16, Norvasc**	0,305	0,082	**-2,447**	**0,002**	-0,201	0,254	4,278	**(0,000)**	5,803	-42,17
**Atovastatin 20mg tabs** (102,103,104)*	**17, Lipitor**	0,349	0,001	**-2,645**	**(0,000)**	-0,114	0,17	7,665	**(0,000)**	9,41	-28,11
**Beclomethasone100mcg/dose inh**	**18, Becotide**	-17,698	0,035	-6,847	0,809	18,412	0,269	164,637	**(0,000)**	76,147	-8,99
	105, Beclate	5,532	0,008	**-32,077**	**(0,000)**	-1,221	0,506	71,608	**(0,000)**	99,268	-32,31
	106, Beceze	0,502	0,683	0,791	0,859	-1,745	0,21	109,915	**(0,000)**	112,425	0,70
	107, Qvar	4,159	0,614	**-62,347**	**0,025**	3,355	0,685	157,942	**(0,000)**	174,578	-35,71
**Cephalexin 250mg caps**	**19, Keflex**	0,78	0,004	**-7,919**	**(0,000)**	-0,752	0,093	5,665	**(0,000)**	9,565	-82,79
(109)*	108, Ranceph	-0,417	(0,000)	**0,735**	**0,003**	**0,413**	**(0,000)**	1,839	**(0,000)**	***-0*,*246***	***-298*,*78***
	110, Cpl-Cephalexin	-0,263	(0,000)	**-1,158**	**(0,000)**	**0,273**	**(0,000)**	2,01	**(0,000)**	1,484	-78,03
**Enalapril 10mg tabs**	**20, Renitec**	-0,56	0	0,159	(0,589	**0,573**	**(0,000)**	3,859	**(0,000)**	1,059	15,01
	111, Pharmapress	-0,413	0	-0,133	0,238	**0,463**	**(0,000)**	2,55	**(0,000)**	0,485	-27,42
	112, Alapren	-0,433	0	**-0,19**	**0,025**	**0,438**	**(0,000)**	2,318	**(0,000)**	1,019	-18,65
	113, Enap	-0,387	0	-0,092	0,42	**0,426**	**(0,000)**	2,08	**(0,000)**	0,919	-10,01
**Fluoxetine 20mg tabs**	**21, Prozac**	0,579	0	**-2,787**	**(0,000)**	**-0,324**	**0,001**	6,021	**(0,000)**	8,916	-31,26
(116)*	114, Lorien	-1,602	0	**0,443**	**0,007**	**1,685**	**(0,000)**	4,085	**(0,000)**	0,881	***50*,*28***
	115, Nuzak	-0,117	0	**-1,458**	**(0,000)**	**0,162**	**(0,000)**	2,763	**(0,000)**	2,178	-66,94
**Gliclazide 80mg tabs**	**22, Diamicron**	0,093	0,004	**-0,311**	**0,01**	-0,055	0,084	0,873	**(0,000)**	1,338	-23,24
	117, Adco-Glucomed	0,012	0,53	**-0,264**	**0,002**	-0,004	0,822	0,817	**(0,000)**	0,877	-30,10
	118, Sandoz Gliclazide	-0,005	0,695	**-0,286**	**(0,000)**	0,002	0,91	0,85	**(0,000)**	0,825	-34,67
	119, Diaglucide	-0,157	0	**-0,1091**	**0,041**	**0,173**	**(0,000)**	1,26	**(0,000)**	0,632	-17,26
**Hydrochlorothiazide 25mg tabs**	**23, Dichloride (No data)**	0,031	0,178			** **	** **	0,742	0,009	0,897	0,00
	120, Ridaq	0,123	0	**-0,635**	**0(0,000)**	**-0,094**	**0,001**	0,693	**(0,000)**	1,062	-59,79
	121, Hexazide	-0,248	0	**0,32**	**0,001**	**0,26**	**(0,000)**	0,645	**(0,000)**	***-0*,*595***	***-53*,*78***
**Ibuprofen 200mg tabs**	**24, Brufen**	0,034	0	**-0,103**	**(0,000)**	**-0,019**	**0,006**	0,419	**(0,000)**	0,589	-17,49
(123)*	122, Inza	-0,016	0	**-0,149**	**(0,000)**	**0,021**	**(0,000)**	0,36	**(0,000)**	0,28	-53,21
	124, Ranfen	-0,015	0	**-0,168**	**(0,000)**	**0,016**	**(0,000)**	0,304	**(0,000)**	0,229	-73,36
**Metformin 500mg tabs**	25, Glucophage	-0,021	0,027	**-0,2**	**(0,000)**	**0,038**	**0,001**	0,606	**(0,000)**	0,501	-39,92
(125, 126, 127)*	128, Sandoz-Metformin	0,021	0,094	**-0,262**	**(0,000)**	**-0,022**	**0,099**	0,465	**(0,000)**	0,57	-45,96
**Metronidazole 200mg tabs**	26, Flagyl	0,195	0	**-0,609**	**(0,000)**	**-0,125**	**(0,000)**	0,721	**(0,000)**	1,696	-35,91
(131)*	129, Metazol	0,01	0	**-0,084**	**(0,000)**	**-0,006**	**(0,000)**	0,087	**(0,000)**	0,137	-61,31
	130, Trichazole	0,006	0,202	**-0,246**	**(0,000)**	**-0,004**	**0,381**	0,326	**(0,000)**	0,356	-69,10
**Nifedipine Retard 10mg tab**	27, Adalat Ret	0,324	0	**-0,632**	**0,003**	**-0,147**	**0,016**	1,788	**(0,000)**	3,408	-18,54
	132, Nifedelat	0,035	0,001	**-0,7**	**(0,000)**	**-0,007**	**0,365**	0,935	**(0,000)**	1,11	-63,06
	133, Cardifen	0,038	0,168	**-0,239**	**0,032**	**-0,049**	**0,095**	1,035	**(0,000)**	1,225	-19,51
**Ranitidine 150mg tabs**	28, Zantac	0,333	0,005	**-0,101**	**0,777**	**0,024**	**0,824**	4,038	**(0,000)**	5,703	-1,77
	134, Ultak	-0,272	0,016	**-0,721**	**0,027**	**0,31**	**0,008**	2,461	**(0,000)**	1,373	-52,51
	135, Histak	-0,304	0	**-1,488**	**(0,000)**	**0,344**	**(0,000)**	3,525	**(0,000)**	2,005	-74,21
	136, CPL Ranitidine	-0,972	0	**-1,009**	**(0,000)**	**0,999**	**(0,000)**	3,377	**(0,000)**	1,433	-70,41
**Sodium Valproate200mg Tab** (137, 138)*	29, Epilim	0,151	0	**-0,307**	**0,016**	**-0,035**	**0,292**	1,344	**(0,000)**	2,099	-14,63

Notes

Int 1(Estimate in 2004) = Cons + (Trend X Years)

Statistically Significant (P<0,05) in BOLD

Regional List With Data = Originals:14, Generics: 26; Total = 40

Change in level = 34 (85%); n = 40

Change in level = 23 (57,5%); n = 40

Regional List with no data* = 17

Regional List total = 57

**Table 3:** Supplementary list interrupted time-series analysis for originator and generic molecules using pricing data from 1999 to 2014 with 2004 as the interruption in the series. statistically significant values (P < 0,05).

INN	Trade Names (Originator in Bold)	Trend	(P value)	Change in level	(P value)	Change in slope	(P value	Constant	(P value)	Int 1	*% Change in level 2004*
**Acyclovir 200mg tabs**	**30, Zovirax**	0,677	**(0,000)**	-0,429	0,422	-0,2	0,187	8,551	**(0,000)**	11,936	-3,59
(140)*	139, Cyclivex	0,047	0,344	**-1,827**	**(0,000)**	0,039	0,442	2,879	**(0,000)**	3,114	-58,67
** **	141, Lovire	0,041	**0,031**	**-1,585**	**(0,000)**	0,027	0,151	2,62	**(0,000)**	2,825	-56,11
** **	142, Adco-Acyclovir	0,072	0,504	-0,297	0,464	-0,165	0,165	2,678	**(0,000)**	3,038	-9,78
**Carbamazepine 200mg tabs**	**31, Tegretol**	0,175	**(0,000)**	**-0,477**	**0,001**	**-0,078**	**0,023**	1,464	**(0,000)**	2,339	-20,39
(144)*	143,Degranol	0,105	**(0,000)**	**-0,294**	**0,001**	-0,04	0,069	0,762	**(0,000)**	1,287	-22,84
** **	145, Sandoz Carbamazepine	0,132	**(0,000)**	**-0,781**	**(0,000)**	**-0,101**	**(0,000)**	0,781	**(0,000)**	1,309	-59,66
**Amox/Clav inj 600mg** (146,147)*	**32, Augmentin**	3,13	**(0,000)**	**-9,99**	**0,001**	**-2,211**	**0,006**	7,841	**(0,000)**	23,491	-42,53
**Digoxin 0,25mg tab**	**33, Lanoxin**	0,021	**(0,000)**	**-0,09**	**(0,000)**	**-0,014**	**0,001**	0,206	**(0,000)**	0,311	-28,94
** **	148, Purgoxin	0,019	**(0,000)**	**-0,055**	**0,001**	-0,004	0,223	0,187	**(0,000)**	0,282	-19,50
**Fluconazole 200mg cap** (149, 150, 151)*	**34, Diflucan**	-0,238	0,729	**-6,626**	**0,013**	**2,738**	**0,004**	50,703	**(0,000)**	49,513	-13,38
**Ketoconazole 200mg tab** (153)*	**35, Nizoral**	2,079	**(0,000)**	**-3,45**	**0,017**	**-1,107**	**0,031**	13,609	**(0,000)**	24,004	-14,37
** **	152, Ketazol	3,519	**0,001**	**-14,796**	**(0,000)**	**-3,239**	**0,002**	10,883	**(0,000)**	21,44	-69,01
**Losartan 50mg tab** (154, 155)*	**36, Cozaar**	-0,017	0,948	-0,096	0,911	-0,276	0,381	5,213	**(0,000)**	5,128	-1,87
**Phenytoin 100mg caps** (156)*	**37,Epanutin**	0,13	**(0,000)**	**-0,43**	**(0,000)**	**-0,055**	**0,028**	0,979	**(0,000)**	1,629	-26,40
**Rifampicin 150mg caps** (157)*	**38, Rimactane**	0,185	**(0,000)**	**-0,555**	**(0,000)**	**-0,132**	**(0,000)**	0,542	**(0,000)**	1,467	-37,83
**Rosuvastatin 10mg tabs** (158, 159, 160)*	**39, Crestor (no data)**	0,239	**(0,000)**	** **	** **	** **	** **	4,551	**(0,000)**	5,746	***0*,*00***
**Ofloxacin 200mg tabs** (162)*	**40, Tarivid**	2,128	**(0,000)**	**-5,167**	**(0,000)**	**-1,113**	**0,004**	10,353	**(0,000)**	20,993	-24,61
** **	161, Tafloc	-0,852	**0,025**	**-4,001**	**(0,000)**	**1,225**	**0,004**	11,688	**(0,000)**	9,984	-40,07
**Aminophylline 250mg inj**	**41, Aminophylline**	2,178	**(0,000)**	**-7,891**	**(0,000)**	**-0,944**	**0,04**	16,003	**(0,000)**	26,893	-29,34
** **	163, SFK Aminophylline	0,857	**(0,000)**	**-4,824**	**(0,000)**	**-0,488**	**(0,000)**	6,608	**(0,000)**	10,893	-44,29
** **	164, Merck A Aminophylline	1,729	**(0,000)**	**-9,478**	**(0,000)**	**-1,588**	**(0,000)**	3,43	**(0,000)**	12,075	-78,49
**Miconazole Nitrate 2% crm** (166)*	**42, Daktarin**	0,526	**(0,000)**	**-0,971**	**0,001**	**-0,269**	**0,003**	2,4	**(0,000)**	5,03	-19,30
** **	165, Covarex	0,098	0,011	**-1,453**	**(0,000)**	-0,068	0,057	1,969	**(0,000)**	2,263	-64,21
**Erythromycin 250mg tabs**	**43, Erythrocin**	0,329	0,323	0,7	0,611	**-1,915**	**0,004**	4,346	**(0,000)**	5,991	11,68
** **	167, Purmycin	0,048	**(0,000)**	**-0,794**	**(0,000)**	**-0,022**	**0,025**	1,097	**(0,000)**	1,289	-61,60
** **	168, Xeramel	0,189	0,104	**-1,003**	**(0,000)**	-0,163	0,161	1,065	**(0,000)**	1,443	-69,51
**Azithromycin 500mg tabs** (169, 170, 171)*	**44, Zithromax**	2,872	**(0,000)**	**-10,698**	**(0,000)**	**-1,595**	**-0,017**	27,625	**(0,000)**	41,985	-25,48
**Cimetidine 200mg tabs**	**45, Lenamet**	-0,22	**(0,000)**	**-0,31**	**0,001**	**-0,238**	**(0,000)**	1,655	**(0,000)**	0,555	-55,86
** **	172, Secadine	-0,311	**(0,000)**	**-0,283**	**0,002**	**0,326**	**(0,000)**	1,443	**(0,000)**	0,51	-55,49
** **	173, Cimlok	-0,19	**(0,000)**	**-0,397**	**0,006**	**0,198**	**(0,000)**	1,608	**(0,000)**	0,658	-60,33
**Lisinopril 10mg tabs** (175, 177)*	46, Zestril	0,187	0,231	**-1,66**	**0,231**	**-0,217**	**0,186**	2,917	**(0,000)**	3,852	-43,09
** **	176, Adco-Zetomax	-0,131	0,02	**-0,528**	**0,003**	**0,178**	**0,003**	2,148	**(0,000)**	1,624	-32,51
**Loratadine 10mg tabs** (178, 179, 180)*	47, Clarityne	0,249	0,267	**-1,013**	**0,23**	**-0,667**	**0,012**	5,342	**(0,000)**	6,587	-15,38
**Ceftazidime I1g/vial inj** (181, 182, 183)*	48, Fortum	10,593	**(0,000)**	**-35,972**	**(0,000)**	**-7,382**	**0,004**	98,727	**(0,000)**	151,692	-23,71
**Isosorbide Mononitrate 20mgT**	49, Ismo	0,377	**(0,000)**	**-1,195**	**(0,000)**	**-0,255**	**(0,000)**	1,153	**(0,000)**	3,038	-39,34
** **	184, Elantan	0,168	**(0,000)**	**-0,531**	**(0,000)**	**-0,097**	**0,003**	0,868	**(0,000)**	1,708	-31,09
**Thyroxine 50mcg tab** (185, 186)*	50, Eltroxin	0,034	0,001	**-0,028**	**0,303**	**-0,004**	**0,614**	0,333	**(0,000)**	0,503	-5,57
** **	174, Adco-cimetidine	-0,064	0,236	**-0,305**	**0,026**	0,045	0,399	1,006	**(0,000)**	0,814	-37,47

Notes

Int 1(Estimate in 2004) = Cons + (Trend X Years)

Statistically Significant (P<0,05) in BOLD

Supplementary list With Data (Originals: 20, Generics: 18) = 38

Change in level = 31 (75,6%)

` Change in slope = 26 (63,4%)

Supplementary List with no Data– 31

Supplementary List Total = 69

The global core in Table 1 contains the data for 43 molecules (14 originator, 29 generics). Of the fourteen (14) original molecules ten (10) showed a statistically significant (P<0.05) change in level with twenty-five (26) of the twenty-eight (28) generics showing a statistically significant change in level (P<0.05). The level change indicated an immediate decrease in the medicine price on the introduction of the regulation in 2004. 65.12% of the molecules showed a statistically significant (P<0.05) change in slope indicating that the policy will continue to benefit medicine prices over time.

Table 2 contains the data for the regional core basket of 40 molecules (14 original and 26 generics). Of the 14 originator molecules 11 showed a statistically significant change in level (P<0.05) with seven showing statistically significant change in slope. Twenty-three (23) of the 26 generics showed a statistically significant change in level (P<0.05) with 16 showing statistically significant change in slope.

In the 38 molecules analysed in the supplementary basket 31 showed statistically significant change in level (75.60%) and 26 (63.4%) showed statistically significant change in slope.

**Fig 1:**
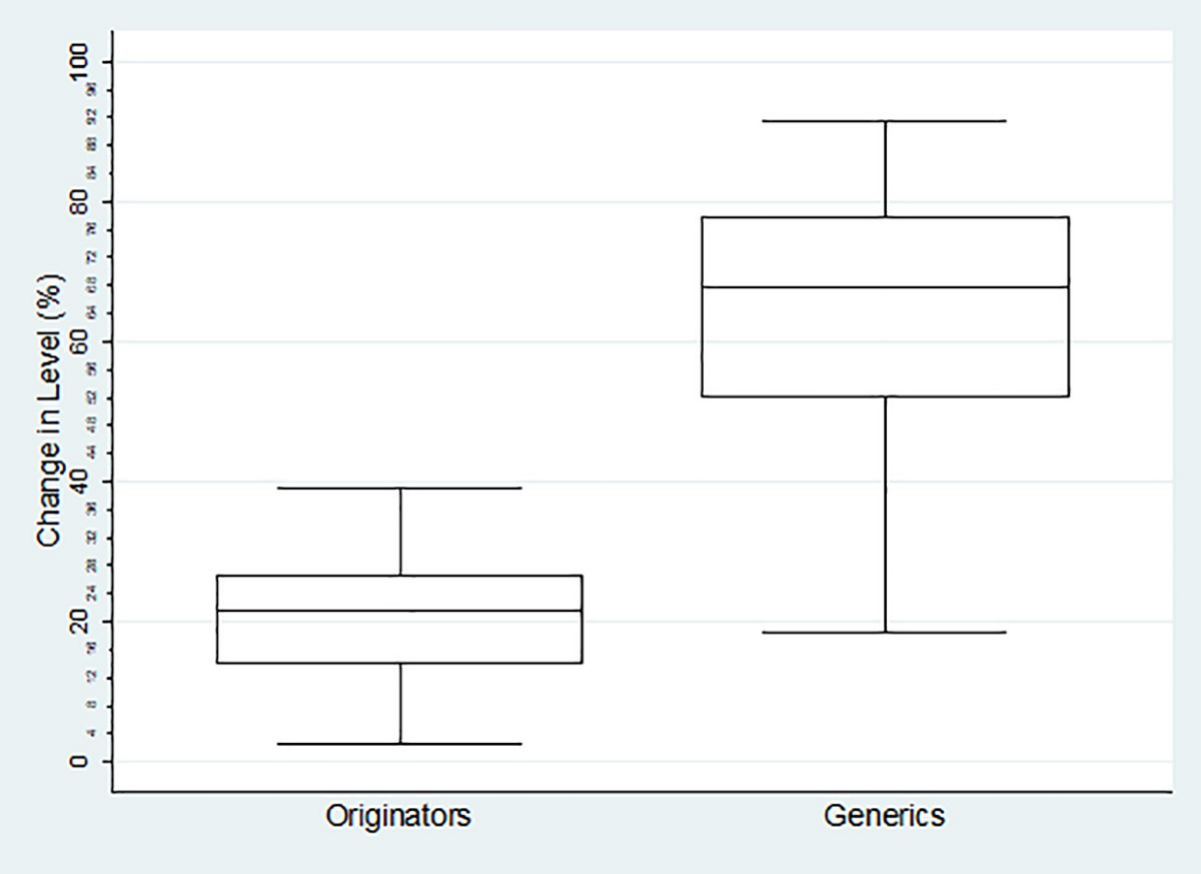
Percentage change in level in the Global Core Basket.

**Fig 2:**
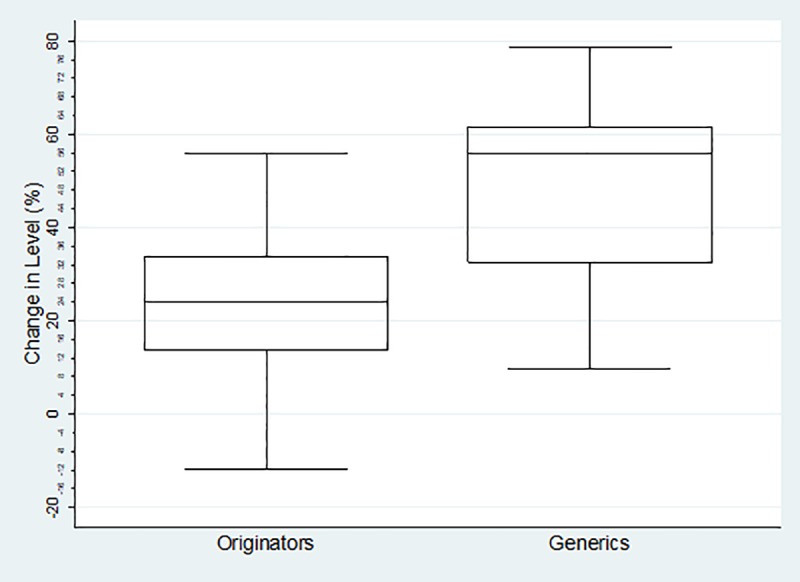
Percentage change in level in the Regional Core Basket.

**Fig 3:**
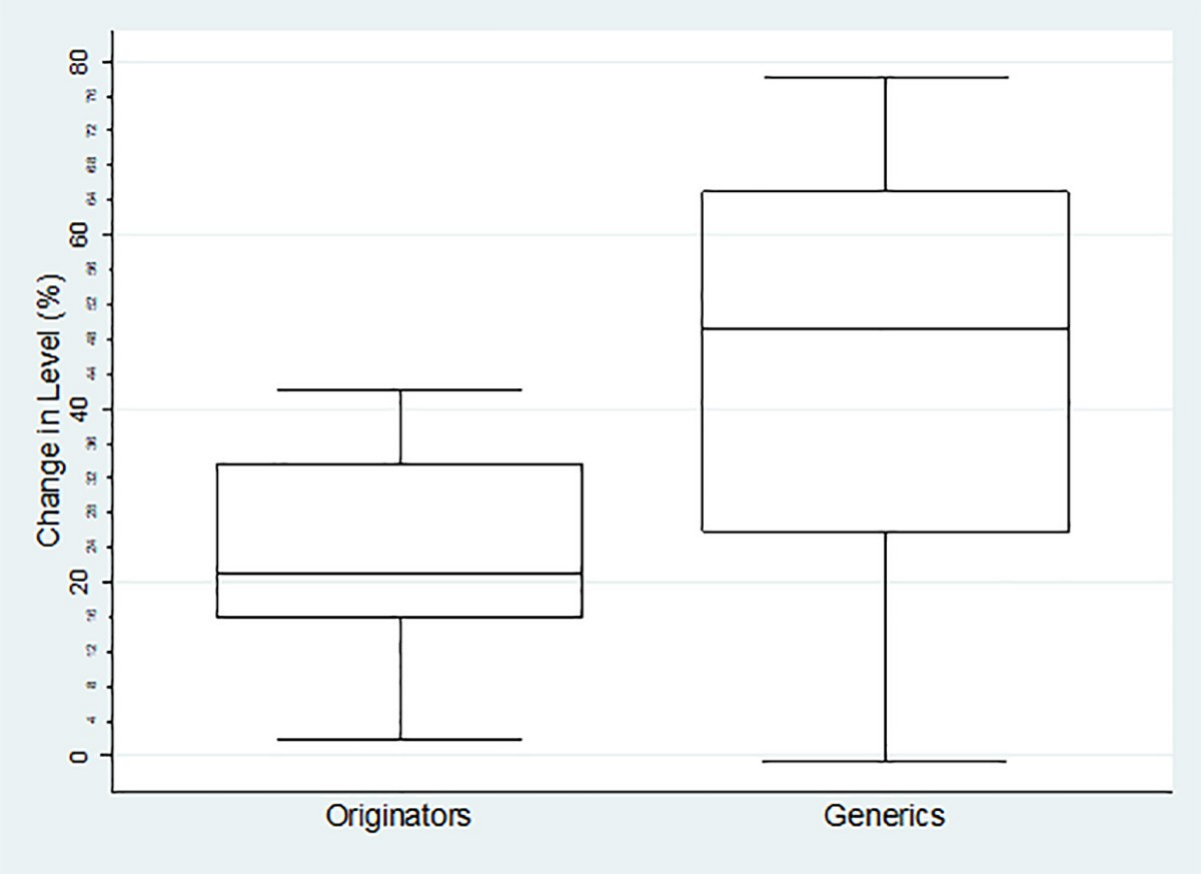
Percentage change in level in the Supplementary Basket.

The boxplots of percentage change in level for each category of medicines are reflected below. For the Global Core (Fig 1) the percentage change ranged from 2.45%-39.12% (mean = 19.87%, SD = 10.62% IQR = 10.2%) for the originator medicines and 18.50%-91.5% (mean = 62.46%, SD = 18.64%, IQR = 24.81%) for their generics. The range for the Regional Core (Fig 2) was 1.77%-42.17% (mean = 23.38%, SD = 12.43%, IQR = 15.65%) for the originator medicines and -0.70%-78.03% (mean = 44.62%, sd = 23.04%, IQR = 37.41%) for their generics. The Supplementary list (Fig 3) was -11.68%-55.86% (mean = 22.97%, SD = 16.26%, IQR = 17.34) for the originator medicines and 9.78%-78.49% (mean = 48.37%, SD = 19.44%, IQR = 27.53%) for their generics. The negative values in the minimum reflects an increase in price (positive change in level), and all calculations excludes outliers.

**Fig 4:**
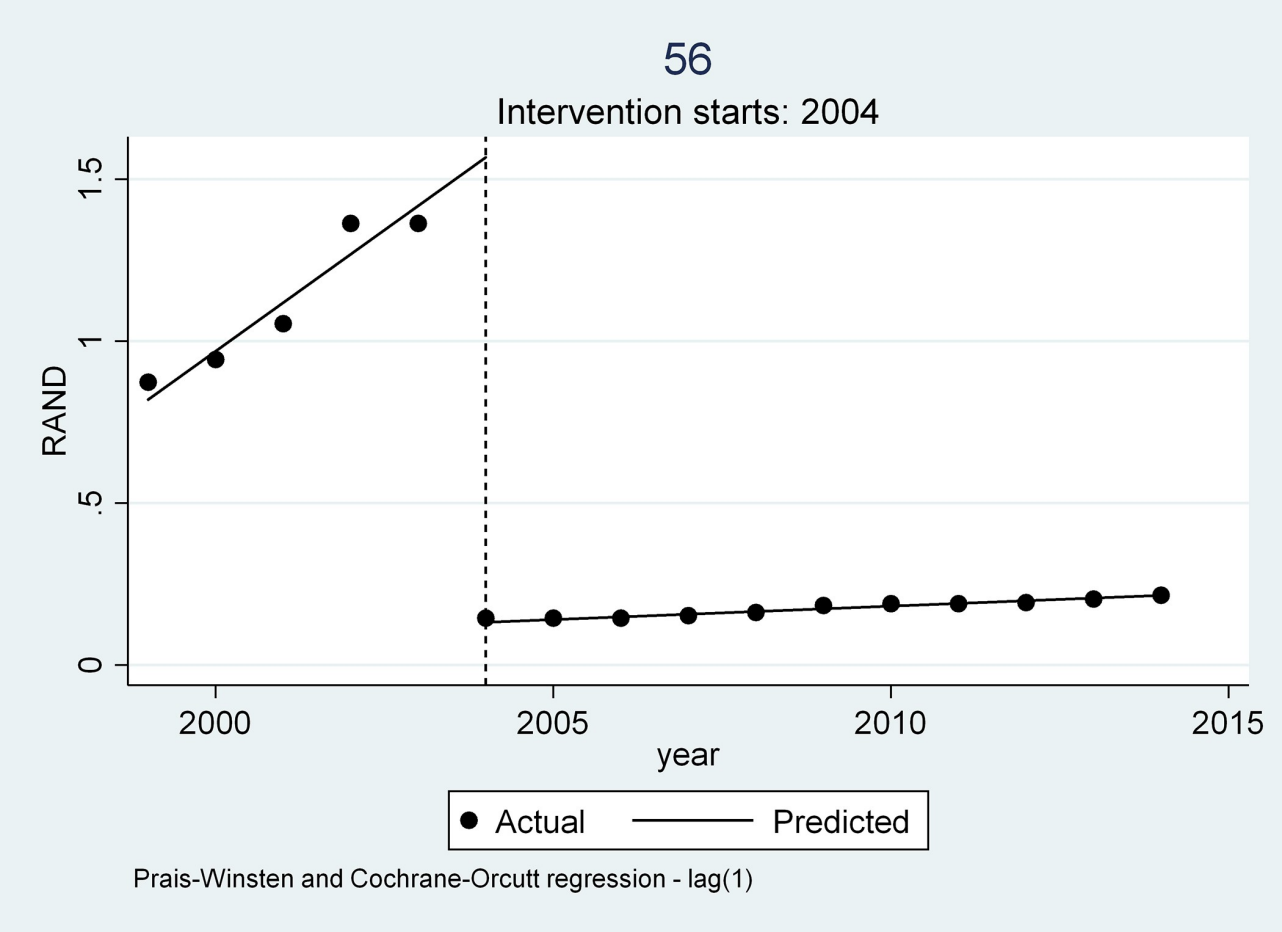
Trend 1 as depicted by Sandoz Glibenclamide.

**Fig 5:**
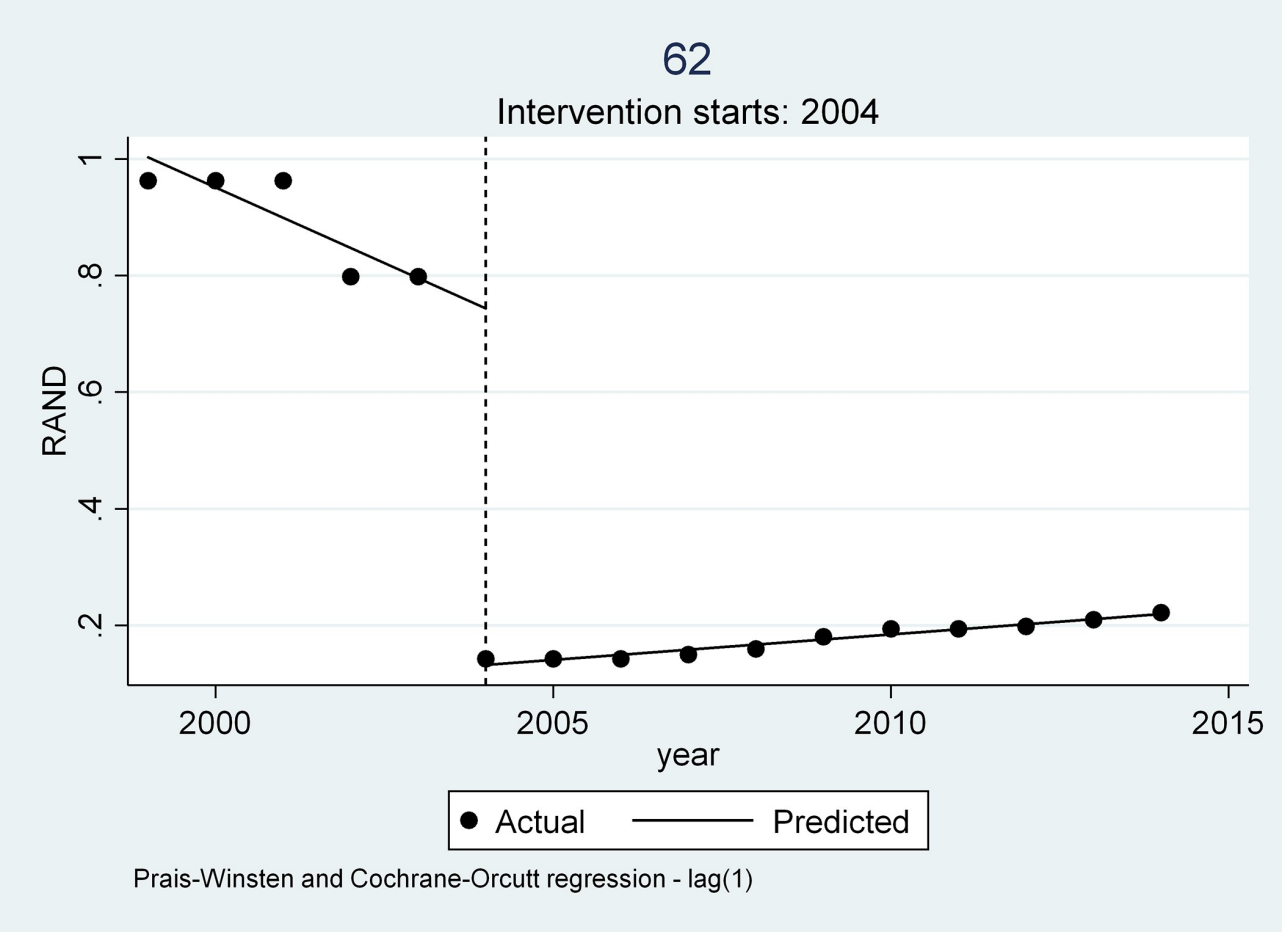
Trend 2 as depicted by Mylan Captopril.

**Fig 6:**
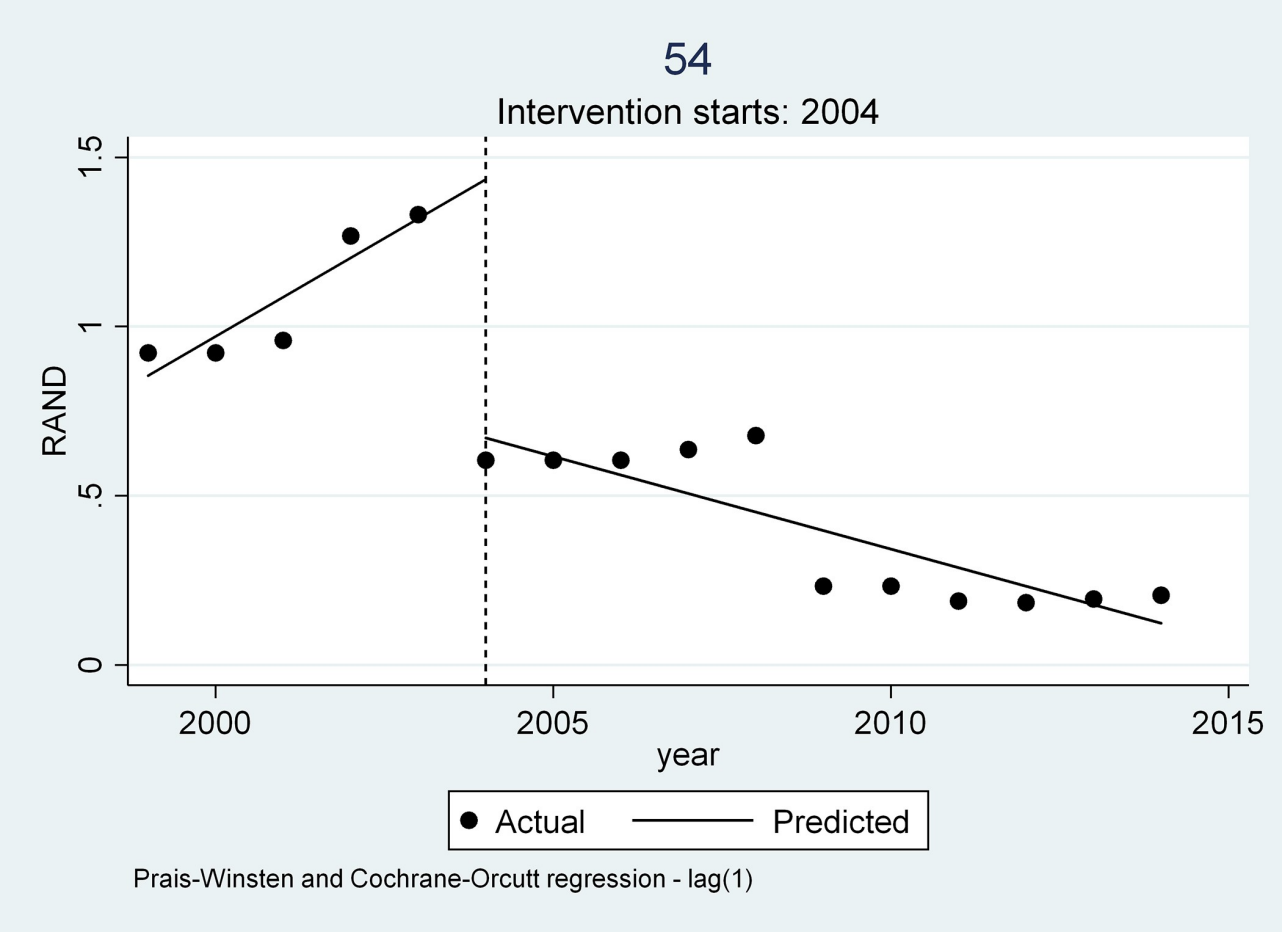
Trend 3 depicted by Glycomin.

Three trends emerged from all the medicines examined as can be seen from Table 4 and Figs 4, 5 and 6.

**Table 4:** Three emerging trends with changes in slope and level.

	Change in Level (P-Value)	95% Conf. Interval	Change in Slope (P-value)	95% Conf. Interval
**Trend 1** **Glibenclamide 5mg tab** 56 Sandoz-Glibenclamid2	-1.436 (0.000)	-1.500 - -1.371	-0.141 (0.000)	-1.59 - -0.28
**Trend 2** **Captopril 25mg tabs** 62. Mylan Captopril	-0.612 (0.000)	-0.668 - -0.555	0.061 (0.000)	0.045–0.076
**Trend 3** **Glibenclamide 5mg tab** 54. Glycomin	-0.764 (0.000)	-1.075 - -0.453	-0.171 (0.001)	-0.260- -0.081

### Trend 1

A visual inspection of the interrupted time series graph (see Fig 4) for Sandoz-Glibenclamide indicates that the medicine prices prior to 2004 showed a year-on-year steady rate of increase (slope 0.150 (P = 0.000), [CI 95% (0.132- -0.167)]. The introduction of the single exit price (SEP) regulations in 2004 saw a price reduction as indicated by the change in level -1.436 (P = 0.000), [CI 95% (-1.500- -1.371)]. In addition, the average rate of increase before the regulation was higher than the average rate of increase after the regulation as indicated in the change in slope (-0.41 (P = 0.000) [CI 95% (-1.59- -0.28)].

The Adjusted R-Squared for Sandoz-Glibenclamide is relatively high at 99.77% indicating that the fitted value closely correlates to the observed prices. The P-Value is 0.000 indicating that there is a high probability of a significant difference in price of the medicine after the policy intervention.

### Trend 2

In trend 2 medicine prices were already decreasing prior to the intervention in 2004 as is evident in the visual inspection with Mylan Captopril 25mg (see Fig 5). The average rate of decrease before intervention of Mylan Captopril was ZAR 0.052 per year(P = 0.000) [CI 95% (-0.067- -0.037)] reflected in the slope. After intervention the medicine saw a price reduction as indicated by the change in level of -0.612 (P = 0.000), [CI 95% (-0.669- -0.555)]. The average price increase after the introduction of the intervention in 2004 as opposed to a decrease is reflected in the change in slope of ZAR 0.061 [CI 95% (0.045–0.076)]. The slope change in Trend 2 indicates that the medicines will lose most of their gains over time.

Twenty-eight (28) of the generic medicines studied in the three baskets followed the pattern in Trend 2. Twenty-six (26) of these medicines showed a further drop in price after the intervention with only two showing an immediate price increase in 2004. Two medicines Cifloc 500mg tablet and Cifran 500mg tablet, the only two available generics to Ciprobay 500mg tablet at the time, showed an increase in 2004 following almost an identical pricing trend to each other during the study period.

### Trend 3

Three (3) generics in the basket, Glycomin 5mg tablet, Aspen Ceftriaxone 1g injectable vial in the global core and Cardifen 10mg tablet in the regional core initially followed those in trend 1 where there was a steady increase in price between 1999 to 2004 with a steep drop in 2004. However, there was a subsequent decrease in price during the 2004–2014 period unlike in trend 1. This average rate of decrease after 2004 to 2014 was unusual as most medicines took the regulated increase offered by the National Department of health. Visual inspection of the ITS graphs for all three molecules reflect that the manufacturing companies may have applied for a price reduction normally related to competition or stock issues.

Trend 3 is illustrated using Glycomin 5mg tablet (Fig 6). The Adjusted R-Squared for Glycomin 5mg tablet is 89.22%. The P-Value is 0.000 indicating that there is a high probability of a significant difference in price of the medicine after the policy intervention. The price reduction of the medicine due to the introduction of the intervention in 2004 is reflected in the change in level -0.764 [CI 95 percentage (-1.075- -0.453)] and the change in slope ZAR 0.171 [CI 95% (-0.260–0.081)].

## Discussion

In the WHO Guidelines on Country Pharmaceutical Pricing Policies [21], it is suggested that a gap exists in the quantitative assessment of the impact of policy change on generic medicines in LMICs. Countries enforcing a pro-generic policy should put in place a monitoring and evaluation programme to track data before and after a policy change using an experimental or quasi-experimental design so that if the policy has not provided the intended result it should be reviewed. [21]

A number of policies exist to promote the use of generics and/or lower medicine prices. Many countries facilitate easy market entry, promote substitution by dispensers, introduce international reference prices, promote competition in the market, and encourage use of generics amongst providers and consumers. [21] One of the key contributors to generic use is the assurance of quality and South Africa performs this adequately through its medicine regulatory authority (previous Medicine Control Council (MCC)- now South African Health Product Regulatory Authorities (SAHPRA)). Generic entry is also encouraged where there is a transparent pricing system.[23]

The price of originator medicines internationally are two and a half (2.5) times more than their lowest priced generics. [24] In LMIC this difference could be more than 10 fold. [25] If we examine the Global Core the difference in price is 4.29 times lower than the originator prior to 2004. Directly after the introduction in 2004 of the SEP the difference between the price of the originator molecule and their cheapest generic showed a 11.1 fold increase in South Africa, in line with Cameron and Laing (2010) [25] suggestion for LMICs. Bangalee, et al. revealed in their study on cardiovascular drugs a 40% difference in prices of generics against the branded versions, [7] and this is confirmed in the pre 2004 comparative in this study (42.9%). This study further suggests that the introduction of the SEP increased this differential, at least in the global core basket, to 111%. This supports the observation by Bangalee and Suleman (2015) that originator companies do not engage in price competition. [7] This may also provide a reason for eight (8) of the fifty (50) originator molecules being withdrawn after 2004.

Veena, et al. in India suggested that branded medicines are 30%-200% more costlier than generics. [26] Cameron, et al in their study of middle to low income countries concluded that for the medicines studied, an average of 9% to 89% could be saved by individual countries in the private sector with the change from originator to lowest-priced generics. [27] A Nigerian national survey of 129 medicine outlets where 34 prescription medicines were investigated consumers paid up to 64 times the international reference price. [28]

Medicines as indicated in trend 2 were already on a downward slope prior to the regulations. This may well be related to pressure on manufacturers to have their medicines listed on preferred formularies with medical scheme payers in the private market or to meet the Maximum Medical Aid Price (MMAP), a common tool used in South Africa to reimburse medicines. Other factors to consider may be the entry of other generics that create competition, or in preparation for the anticipated regulatory changes, which was in discussion in South Africa a few years prior to the 2004 implementation when draft regulations were published some seven years earlier. [29]

While most medicines post the intervention took the Government regulated prices increases on a yearly basis, some as in Trend 3 tended to have an average decrease in price over the study period. This was also true for medicines in the data set that could not be fully assessed because of insufficient pre-2004 data (reflected in the supplementary files on the ITSA analysis). Manufacturers of these medicines opted to take one or more voluntary price decreases during the study period. This may have been necessary to fall in line with other similar priced molecules especially if their initial declaration/disclosure prior to 2004 was an overestimation compared to their competitor, introduction of competing medicines in the same generics, stock issues such as over production and short expiry, or pressure in the reimbursement models from medical scheme.

Both generic and originator medicines in the study showed an immediate price decrease in most medicines, a lower yearly increase as compared to yearly increases prior to the regulations and a possible saving due to price reduction over the study period. This is in direct contrast to the Moreno–Torres [30] which concluded that twelve of sixteen pricing interventions introduced in Spain were not effective even in the short term and four were not impactful. This study indicates that the SEP regulation had an impact on both originator and generic medicine pricing in South Africa immediately after introduction and continued over the study period.

There is no doubt that generic penetration creates a saving in any healthcare system but this is dependent on the price levels that the generic is set at and the differential between the originator and generic medicine. [11] Seeley and Kanavos in their examination of seven OECD countries (United States of America(US), France, Germany, Italy, United Kingdom (UK) and Canada) suggested that generic penetration varies significantly and could be improved especially in Italy and France where it appears to be the lowest. [11] Spain and Canada exhibited average levels while the US, UK and Germany showed the highest levels of generic penetration. [11]

According to Kaplan et al (2016) [31], many European countries set the price of a generic at a specific percentage lower than the originator product, and indicate that countries with generic link policies have lower prices compared to countries that do not. Vogler et al. [32] investigated prices of medicines that were likely to contribute to high expenditure for the public payers in high-income countries. Information on the ex-factory price data of 30 medicines in 16 European countries was collected in April 2013. There were considerable differences in medicine prices, with 53% of the medicines surveyed having a unit ex-factory price (median) above 200 Euro. The price differences between the highest-priced country and lowest-priced country ranged between 25% and 100% for two-thirds of the medicines. The mainly low-priced medicines had higher price differential up to 251%.

Generic product sales accounted for 30% of the total pharmaceutical sales in South Africa in 2012. [33] Generic utilization rates (generic items claimed as a percentage of total items claimed from one pharmacy benefit manager in South Africa) in 2013 reached 54.5% in the private sector. [34] Vogler, [35] and Maisonneuve, [36] suggested that generic use can be attributed to countries policy implementation e.g. number of generics, prescribing practices and market structures. Strict regulation of medicine prices may contribute to lower penetration of generic medicines into markets. [37] This may be due to the reduced profitability and the inability of generics to cover their cost of market entry. [38]

The impact of the combination of the SEP policy, with the mandatory offer of generic policy is largely unknown, in terms of both price transparency and generic penetration. There is clear indication in this study that the SEP regulations had a greater impact on generic medicines than it did on originators. Using the global core as an example, the basket of generic molecules showed a markedly better average percentage decrease in level than the originator basket; showing a 42.66% difference. Seventy-five percent of the generic molecules decreased in price by more than 52.32%, while twenty-five percent of the generic molecules showed an even greater decrease of 77,13%. Half of the generic molecules showed a decrease in price between 52.33–77.13%, while the bulk of the originator molecules only showed a decrease of between 14.79–25%, indicating that the generic basket performed much better with the implementation of the 2004 price change regulations. This is also true for both the Regional Core and the Supplementary baskets as reflected in Figs 2 and 3 which reinforces the observation that originator companies do not engage in price competition. [7]

Certain limitations of this study must be considered. The first is the limited data available prior to implementation of the regulations. Bernal, et al. [39] suggest that there are “no fixed limits regarding the number of data points”. The power depends on “various other factors, including distribution of data points before and after the intervention, variability within the data, strength of effect, and the presence of confounding effects such as seasonality”. [27] If the data was done every quarter it would have produced 20 data points over 5 years. Medicine prices in South Africa tend to be stable year-on-year. In viewing the data in the database, we found that the price was stable over four quarters in majority of the medicines. Hence the need to select a single reference point and access the price at that point.

In inspecting the visual results, a recommendation by Bernal, et al., it can be seen that the trend before intervention does not show drastic changes. There is also a clear differentiation between the pre- and the post-intervention period with a well-defined period of implementation- in this case an immediate change. [27] The last limitation is the linear trend assumed by the segmented regression model that was used. [16]

## Conclusion

Further studies need to be done to determine availability and access [40] and possible negative impact of this type of pricing model. Price comparative to international markets may be required to benchmark the introduction price and this could, especially in the originator market, influence the price setting of the SEP. After 14 years of pricing regulations more studies need to be performed on reasons for the introduction of the many generics after 2004, setting of the SEP prices and its international comparisons, influence of reference pricing in the private market, the price of medicines in a single market system as compared to the private/public system in South Africa.

Using the Interrupted Time Series in this study we can conclude that the data reflects a decrease in medicine prices with possible savings having been achieved through the introduction of the SEP regulation in both the originator and generic markets in the private sector in majority of medicines. Despite the limitations highlighted under discussion this study provides evidence of the impact of medicine pricing intervention from a middle–income country, and useful lessons can be drawn by other developing countries looking at introducing medicine price controls.

## Supporting information

S1 Table Global core list data. (XLSX)

S2 Table Regional core list data. (XLSX)

S3 Table Supplementary list data. (XLSX)

## References

[1] Antoñanzas F, Terkola R, Overton PM, Shalet N, Postma M. Defining and Measuring the Affordability of New Medicines: A Systematic Review. Pharmacoeconomics. 2017;35(8):777–91. 10.1007/s40273-017-0514-4 28477220

[2] Carone G, Schwierz C, Xavier A. Cost-containment policies in public pharmaceutical spending in the EU [Internet]. Economic and Financial Affairs. 2012 Available from: 10.2765/27111

[3] Aitken M, Machin C, Troein P. Understanding the pharmaceutical value chain. Pharm Policy Law [Internet]. 2016;18(1–4):55–66. Available from: http://www.medra.org/servlet/aliasResolver?alias=iospress&doi=10.3233/PPL-160432

[4] Council for Medical Schemes. CMS Annual Report 1995 [Internet]. 1995. Available from: https://www.medicalschemes.com/files/Annual Reports/CMS Annual Report 1995.pdf

[5] National Department of Health South Africa. National Drug Policy for South Africa Table of contents. 1996.

[6] Republic of South Africa. Medicines and Related Substances Control Amendment Act (Act 90 of 1997). 18505 South Africa: Government Gazette; 1997.

[7] Bangalee V, Suleman F. Has the increase in the availability of generic drugs lowered the price of cardiovascular drugs in South Africa? Heal SA Gesondheid. 2016;21:60–6.

[8] National Department of Health South Africa. Regulations relating to a transparent pricing system for medicines and scheduled substances [Internet]. NDoh; 2004 Available from: https://www.gov.za/sites/www.gov.za/files/26304.pdf

[9] Bangalee V, Suleman F. Towards a transparent pricing system in South Africa: trends in pharmaceutical logistics fees.

[10] International Federation of Pharmaceutical Manufacturers. The Pharmaceutical Industry and Global Health. 2017.

[11] Seeley E, Kanavos P. Pharmaceutical Policy: cost containment and its impact [Internet]. 2008 p. 18–22. Available from: http://www.euro.who.int/__data/assets/pdf_file/0003/80445/Eurohealth14_2.pdf

[12] Cambourieu C, Pomey M, Cambourieu C. Generic Drug Pricing Policy in Quebec. 2013.

[13] Hollis A. Generic Drug Pricing and Procurement: A Policy for Alberta. Univ Calgary Sch Policy Stud Res Pap. 2008;2(1).

[14] Hollis A, Grootendorst P. Canada’s New Generic Pricing Policy: A Reasoned Approach to a Challenging Problem. Healthc Policy [Internet]. 2015 8;11(1):10–4. Available from: http://www.ncbi.nlm.nih.gov/pmc/articles/PMC4748362/ 26571465 PMC4748362

[15] Hassali MA, Alrasheedy AA, McLachlan A, Nguyen TA, AL-Tamimi SK, Ibrahim MIM, et al The experiences of implementing generic medicine policy in eight countries: A review and recommendations for a successful promotion of generic medicine use. Saudi Pharm J [Internet]. 2014;22(6):491–503. Available from: 10.1016/j.jsps.2013.12.017 25561861 PMC4281627

[16] Wagner AK, Soumerai SB, Zhang F, Ross-Degnan D. Segmented regression analysis of interrupted time series studies in medication use research. J Clin Pharm Ther. 2002;27(4):299–309. 12174032 10.1046/j.1365-2710.2002.00430.x

[17] Gray A, Suleman F. Pharmaceutical Prices in South Africa [Internet]. Babar Z-U-D, editor. Pharmaceutical Prices in the 21st Century. Cham: Springer International Publishing; 2015 251–265 p. Available from: http://www.scopus.com/inward/record.url?eid=2-s2.0-84943386681&partnerID=tZOtx3y1

[18] South Africa N. National Department of Health. South African medicine price registry. Database of medicine prices.

[19] StataCorp. Stata Release 13 [Internet]. Vol. 161–user N. StataCorp LP; 2013 Available from: https://www.stata.com/manuals13/u.pdf

[20] WHO, HAI Global, WHO; HAI. Measuring medicine prices, availability, affordability and price components [Internet]. Vol. 2nd Editio, World Health Organisation 2008 Available from: http://www.who.int/medicines/areas/access/medicines_prices08/en/

[21] World Health Organization. WHO Guideline on Country Pharmaceutical Pricing Policies. WHO [Internet]. 2015;134 Available from: http://apps.who.int/iris/bitstream/10665/153920/1/9789241549035_eng.pdf

[22] Xiphu L, Mpanza N. Medicine prices survey in the Gauteng province in South Africa [Internet]. 2004 Available from: http://www.haiweb.org/medicineprices/surveys/200411ZAG/survey_report.pdf

[23] Vogler S, Zimmermann N, Leopold C, de Joncheere K. Pharmaceutical policies in European countries in response to the global financial crisis. South Med Rev. 2011;4(2):22–32. 10.5655/smr.v4i2.1004 PMC3471176 23093885

[24] Cameron A, Ewen M, Ross-Degnan D, Ball D, Laing R. Medicine prices, availability, and affordability in 36 developing and middle-income countries: a secondary analysis. Lancet. 2009;373(9659):240–9. 10.1016/S0140-6736(08)61762-6 19042012

[25] Cameron A, Laing R. Cost savings of switching private sector consumption from originator brand medicines to generic equivalents. World Heal Report, Backgr Pap 35. 2010;11.

[26] R. V. Generic prescriptions and dispensing in India—problems and solutions—a study. World J Pharm Res [Internet]. 2017 9 1;6(09):414–29. Available from: http://wjpr.net/dashboard/abstract_id/7646

[27] Cameron A, Mantel-Teeuwisse AK, Leufkens HGM, Laing RO. Switching from originator brand medicines to generic equivalents in selected developing countries: How much could be saved? Value Heal [Internet]. 2012;15(5):664–73. Available from: 10.1016/j.jval.2012.04.004 22867775

[28] Auta A, Bala ET, Shalkur D. Generic medicine substitution: A cross-sectional survey of the perception of pharmacists in north-central, Nigeria. Med Princ Pract. 2013;23(1):53–8. 10.1159/000355473 24217185 PMC5586836

[29] Deroukakis M. Mandatory generic substitution. 2007;97(1):63–4. 17378285

[30] Moreno-Torres I, Puig-Junoy J, Raya JM. The impact of repeated cost containment policies on pharmaceutical expenditure: experience in Spain. Eur J Heal Econ. 2011;12(6):563–73. 10.1007/s10198-010-0271-1 20809092

[31] Kaplan W, Wirtz V, Nguyen A, Ewen M, Vogler S, Laing R. Policy Options for Promoting the Use of Generic Medicines in Low- and Middle-income Countries [Internet]. March 2016 Available from http://haiweb.org/wp-content/uploads/2017/02/HAI_Review_generics_policies_final.pdf

[32] Vogler S, Zimmermann N, Babar Z. Price comparison of high-cost originator medicines in European countries, Expert Review of Pharmacoeconomics & Outcomes Research, 201;717:2, 221–230. 10.1080/14737167.2016.1223543 27658050

[33] Barron P. African private pharmaceutical market [Internet]. University of Pretoria; 2014 Available from: https://repository.up.ac.za/bitstream/handle/2263/41894/Barron_Management_2013.pdf?sequence=1

[34] Mediscor. Mediscor Medicines Review 2013 [Internet]. 2013 Available from: http://www.mediscor.net/MMR/Mediscor%2520Medicines%25.20Review%25202013.pdf.

[35] Vogler S. The impact of pharmaceutical pricing and reimbursement policies on generics uptake: implementation of policy options on generics in 29 European countries─an overview. Generics Biosimilars Initiat J [Internet]. 2012;1(2):93–100. Available from: http://www.gabi-journal.net/the-impact-of-pharmaceutical-pricing-and-reimbursement-policies-on-generics-uptake-implementation-of-policy-options-on-generics-in-29-european-countries─an-overview.html

[36] Maisonneuve CD La, Martins JO. Public spending on health and long-term care: a new set of projections. OECD Econ Policy Pap [Internet]. 2013;6(06):1–39. Available from: http://www.oecd.org/eco/growth/Health FINAL.pdf

[37] Danzon PM, Chao L. Does Regulation Drive Out Competition in Pharmaceutical Markets? J Law Econ [Internet]. 2000;43(2):311–58. Available from: http://www.journals.uchicago.edu/doi/10.1086/467458

[38] Dias V, Henry D, Searles A. MDS-3: Managing Access to Medicines and Health Technologies. Manag Sci Heal [Internet]. 2012;Chapter 9. Available from: http://www.msh.org/resource-center/ebookstore/copyright.cfm.%5Cnwww.mds-online.org

[39] Bernal JL, Cummins S, Gasparrini A. Interrupted time series regression for the evaluation of public health interventions: a tutorial. Int J Epidemiol [Internet]. 2017;46(1):348–55. Available from: 10.1093/ije/dyw098 27283160 PMC5407170

[40] Vernon JA, Santerre RE. Assessing consumer gains from a drug price control policy in the U.S. South Econ J [Internet]. 2005;73:233–45. Available from: http://www.nber.org/papers/w11139%0ANATIONAL

